# The flowering of SDP chrysanthemum in response to intensity of supplemental or night-interruptional blue light is modulated by both photosynthetic carbon assimilation and photoreceptor-mediated regulation

**DOI:** 10.3389/fpls.2022.981143

**Published:** 2022-09-16

**Authors:** Jingli Yang, Jinnan Song, Byoung Ryong Jeong

**Affiliations:** ^1^ Department of Horticulture, Division of Applied Life Science (BK21 Four Program), Graduate School of Gyeongsang National University, Jinju, South Korea; ^2^ Institute of Agriculture and Life Science, Gyeongsang National University, Jinju, South Korea; ^3^ Research Institute of Life Science, Gyeongsang National University, Jinju, South Korea

**Keywords:** photoperiodic response, light intensity, supplemental blue light, night-interruptional blue light, photosynthetic efficiency, carbohydrate accumulation, photoreceptors, florigen or anti-florigen genes

## Abstract

The photoreceptor-mediated photoperiodic sensitivity determines the obligate short-day flowering in chrysanthemum (*Chrysanthemum morifolium* Ramat.) when the night length is longer than a critical minimum, otherwise, flowering is effectively inhibited. The reversal of this inhibition by subsequent exposure to a short period of supplemental (S) or night-interruptional (NI) blue (B) light (S-B; NI-B) indicates the involvement of B light-received photoreceptors in the flowering response. Flowering is mainly powered by sugars produced through photosynthetic carbon assimilation. Thus, the light intensity can be involved in flowering regulation by affecting photosynthesis. Here, it is elucidated that the intensity of S-B or NI-B in photoperiodic flowering regulation of chrysanthemums by applying 4-h of S-B or NI-B with either 0, 10, 20, 30, or 40 μmol·m^−2^·s^−1^ photosynthetic photon flux density (PPFD) in a 10-h short-day (SD10) [SD10 + 4B or + NI-4B (0, 10, 20, 30, or 40)] or 13-h long-day (LD13) condition [LD13 + 4B or + NI-4B (0, 10, 20, 30, or 40)] provided by 300 ± 5 μmol·m^−2^·s^−1^ PPFD white (W) LEDs. After 60 days of photoperiodic light treatments other than the LD13 and LD13 + NI-4B (40), flowering with varying degrees was observed, although the SD10 gave the earliest flowering. And the LD13 + 4B (30) produced the greatest number of flowers. The flowering pattern in response to the intensity of S-B or NI-B was consistent as it was gradually promoted from 10 to 30 μmol m^−2^ s^−1^ PPFD and inhibited by 40B regardless of the photoperiod. In SD conditions, the same intensity of S-B and NI-B did not significantly affect flowering, while differential flowering inhibition was observed with any intensity of NI-B in LDs. Furthermore, the 30 μmol·m^−2^·s^−1^ PPFD of S-B or NI-B up-regulated the expression of floral meristem identity or florigen genes, as well as the chlorophyll content, photosynthetic efficiency, and carbohydrate accumulation. The 40B also promoted these physiological traits but led to the unbalanced expression of florigen or anti-florigen genes. Overall, the photoperiodic flowering in response to the intensity of S-B or NI-B of the SDP chrysanthemum suggests the co-regulation of photosynthetic carbon assimilation and differential photoreceptor-mediated control.

## Introduction

The change from the vegetative to the regenerative stage is perhaps the main formative stage in the life cycle of plants. The timing of blooming during the year, which is a significant versatile attribute that unequivocally impacts reproductive fitness, is impacted by both endogenous and ecological variables. Changes in photoperiod (day length) are among the most significant and solid signals for plants to reproduce in favorable seasons. In 1920, Garner and Allard showed that some plant species bloomed depending on the changes in day length and depicted this peculiarity as “photoperiodism” (Garner and Allard, 1920). Plants are grouped by their photoperiodic reactions as short-day plants (SDPs), in which blooming happens when the night duration is longer than a critical minimum; long-day plants (LDPs), in which blossoming happens when the day duration turns out to be longer than some crucial length; and day-neutral plants (DNPs). According to the photoperiodic reactions, there are obligate (qualitative) and facultative (quantitative) types within the SDPs and LDPs. The particular photoperiod is absolute for obligate-type plants to make responses. Chrysanthemum is a kind of obligate SDP. *Chrysanthemum morifolium* is one of 30 flowering species in the *Chrysanthemum* genus of the *Asteraceae* family (Jeong et al., 2012). The flower bud will be induced when the night length ≈ 12 h or more, while the specific minor differences in the photoperiod sensitivity of flower formation are species- or cultivar-depending (McMahon and Kelly, 1999; Higuchi et al., 2013).

Light is well known to functionally regulate flowering in several plant species. So how do plants sense and transmit light signals to induce flowering? It soon became clear that leaves perceive the day-length signals (Knott, 1934). Multiple photoreceptors are mainly located in plant leaves to sense the environmental light signals and seasonal changes in photoperiod, which they take as signals to flower. Different photoreceptors control plant growth and development differently, thus this is the case for the photoperiodic-perception process (Davis, 2002). Both cryptochromes and phytochromes abundance relies on light, which shows the importance of the photoreceptors regarding determining day length (Somers et al., 1998). Additionally, photoreceptors are also sensitive to light quality. Phytochrome B (PHYB) promotes early flowering in *Arabidopsis* in response to low-red/far-red (R/FR) along with PHYD and E (Franklin et al., 2003), however, this response of PHYB in shade avoidance is distinct from photoperiodism, which is inhibitory (Mockler et al., 2003). Furthermore, cryptochromes mediate plant responses to B light and UV-A. Two members of the cryptochromes (CRY1 and 2) are present in *Arabidopsis* and led to early flowers, indicating that cryptochromes play a role to promote flowering (Mockler et al., 1999). Some researchers believed that light does not separately regulate the photoperiod sensitivity in plants, it should be accomplished by combining endogenous circadian rhythms (Biinning, 1936; Pittendrigh and Minis, 1964). Later confirmed that light directly modulated the activity of clock-controlled genes (CCGs), and controlled the circadian phase of the CCGs by resetting the circadian clock (Yanovsky and Kay, 2003; Kobayashi and Weigel, 2007). Overall, these photoreceptors influence flowering by detecting the specific light quality and directing light input to the circadian clock, as well as by altering the protein stability of CONSTANS (CO), a key activator of FLOWERING LOCUS T (FT) (Somers et al., 1998; Valverde et al., 2004).

After plants receive the light signals, a transmissible factor, florigen, is synthesized in leaves, which is the vector of received photoperiodic signals. And the flowering improvement “florigen” was proposed by Chailakhyan in 1936, through a chrysanthemum experiment (Chailakhyan, 1936). Currently, many studies have reported that FT and its orthologs are synthesized in several species’ leaves, which act as florigens (Kobayashi and Weigel, 2007; Zeevaart, 2008; Turnbull, 2011; Mcgarry and Ayre, 2012). In *Arabidopsis*, FT moves into the shoot apical meristem (SAM) through the phloem and in there structures a transcriptional complex with FD, an essential leucine zipper (bZIP) record factor; subsequently activates the *FRUITFULL* (*FUL*) and *APETALA 1* (*AP1*), the transcription of floral regulator genes, leading to flowering (Abe et al., 2005; Wigge et al., 2005). *FT* encodes a phosphatidylethanolamine–binding protein-like (PEBP-like) small protein, that is florigen. The PEBP family has developed both the activators and repressors of blooming. There are five more members of the *FT* gene family in *Arabidopsis*: *TERMINAL FLOWER 1* (*TFL1*), *MOTHER OF FT AND TFL1* (*MFT*), *BROTHER OF FT AND TFL1* (*BFT*), *TWIN SISTER OF FT* (*TSF*), and *Arabidopsis thaliana CENTRORADIALIS homolog* (*ATC*). Based on numerous studies of the FT family: FT and TSF work as floral activators (Kobayashi et al., 1999; Kardailsky et al., 1999; Yamaguchi et al., 2005) while TFL1, ATC, and BFT act as floral repressors (Bradley et al., 1997; Mimida et al., 2001; Yoo et al., 2010), moreover, MFT is related to seed germination (Xi et al., 2010). Additionally, the expression of ATC and TFL1 are observed in vasculature tissue and shoot apex, respectively, even though they are known as non-cell-autonomous (Conti and Bradley, 2007; Huang et al., 2012). Floral repressors also known as an antiflorigenic stimulus are synthesized in leaves too (Thomas and Vince-Prue, 1996), which has clearly confirmed by the classical physiological experiments of the flowering inhibition experiment with grafted leaves in tobacco cultivars under non-floral-inductive light-duration environments (Lang et al., 1977). And the anti-floral factors were also observed in chrysanthemum leaves under flowering unfavorable day-length conditions (Tanaka, 1967). These studies strongly support the idea that the balanced signals of florigens and anti-florigens synthesized in leaves might be involved in differential photoreceptor-mediated regulation in photoperiodic flowering in plants.

Light intensity-related photosynthetic efficiency and carbon assimilation also affect plant flowering, which is associated with altered carbon and nitrogen metabolism (Eckardt et al., 1997; Walters et al., 2004). Low light generally delays the first flowering period of plants, prolongs the flowering period, and decreases the flowering index, which has been studied mostly in tomatoes and some flowering plants (Fc, 1997). The reduced accumulation of nutrients in tomato plants under low light and the poor ability of reproductive growth to compete for photosynthetic products lead to delayed floral bud differentiation, increased flowering nodes, reduced bud quality, and flower number (Kinet, 1977). Additionally, the effect of light intensity on the flowering time of plants under strong light varies widely, and it is generally believed that increasing light intensity has a negative effect on flowering. Since light intensity is related to the photosynthesis and assimilation efficiency of plants, and the photoreceptors of plants are chlorophyll and other photosynthetic pigments, it is possible that the regulation of flowering time is related to changes in photosynthetic and carbon assimilation efficiency. One study found that the delayed flowering in *phya* mutants under low irradiation but not high irradiation, suggesting that the function of *PHYA* might be indirectly mediated through photosynthesis in *Arabidopsis*, however, it still needs further study (Bagnall and King, 2001). Moreover, the flowering of parthenogenic short-day plants was studied at different light intensities (42, 45, 92, and 119 μmol·m^−2^·s^−1^ PPFD), and it was found that the plants flowered earliest at low irradiance (42 μmol·m^−2^·s^−1^ PPFD) (Jalal-Ud-Din et al., 2013). Hence, light intensity involves flowering regulation by affecting photoperiodic carbon assimilation which might be mediated indirectly by *PHYA* at the same time.

Since the technical skills are widely used in chrysanthemum cultivation, the photoperiodic limitation in its flowering time is lifted by blackouts or artificial colorful lighting, day-length extension, or night break (NB) to fulfill the need for marketable flowers consistently. Light supplementation might appear as valuable light in a foundation of regular light, or extra light that expands the day length (Zheng et al., 2018). NB interrupts the continued dark duration with lighting, thus making modulated LD environments (Yamada et al., 2008; Park et al., 2015). Higuchi et al. (2012) revealed the effect of B light on photoperiodic regulation of flowering in chrysanthemums, which found that for plants grown under SD conditions with white (W) light, NB treatment with monochromatic red (R) light was effective in inhibiting flowering, while the monochromatic B light or far red (FR) light was less effective in inhibiting flowering. And the 4-h low level of B light supplied either at the supplementary or NI in SD conditions all flowered and did no significant differences when compared with the normal SD environment (Park and Jeong, 2020). Especially in LD conditions, the non-flowered plants were flowered after being treated with 4-h low-intensity of S-B or NI-B, just deferred flower bud formation (Park and Jeong, 2019; Park and Jeong, 2020). However, the extension of the natural sunlight during the first 11 h of the photoperiod with either R or B sole light, inhibited flowering in *Chrysanthemum morifolium* (SharathKumar et al., 2021). Thus, there is the cultivar specific- or other subtle details- depending (such as intensity, photoperiod, supplementary, or NB) on the flowering response to the B light.

Here, we determined the photoperiodic response of SDP *Chrysanthemum morifolium* to various intensities of S-B or NI-B by setting the experiment with the pot plants of the ‘Gaya Glory’ cultivar under 18 different photoperiodic light treatments. Our results demonstrated that the photoperiodic flowering of chrysanthemums by the co-regulation of sugar accumulation produced by photosynthetic carbon assimilation and the expression of florigen and anti-florigen genes mediated by differential photoreceptors.

## Materials and methods

### Plant materials and growth conditions

The pot experiment was conducted in a closed-type plant factory (770.0 cm long × 250.0 cm wide × 269.5 cm high, Green Industry Co. Ltd., Changwon, Korea) at Gyeongsang National University, Jinju, Korea, in early September of 2021. The ornamental species chrysanthemum (*Chrysanthemum morifolium* Ramat.) ‘Gaya Glory’, a qualitative SDP, was selected as experimental material in this study. The rooted cuttings with 8 ± 1 leaves per plant were obtained from the Flowers Breeding Research Institute, Gyeongnam Agricultural Research & Extension Services, Changwon, Gyeongnam, Korea and separately transplanted into 10 cm plastic pots with commercial medium (BVB Medium, Bas Van Buuren Substrates, EN-12580, De Lier, The Netherlands), one plant per pot. After planting, plants were moved to this closed–type plant factory and acclimated to 23/18°C (light/dark), 70 ± 10% RH, and 270 ± 5 μmol·m^−2^·s^−1^ PPFD supplied with F48T12-CW-VHO fluorescent lamps (Philips Co., Ltd., Eindhoven, The Netherlands). CO_2_ was provided by a compressed gas tank to timely supplement plant photosynthesis and maintained a concentration of 350 ± 50 parts per million (PPM) through an electrolyte CO_2_ sensor (Model No. GMT220 Carbocap, Vaisala) monitored online. The air circulation system here was installed with the fans evenly and the conditioned air could blow horizontally into the developing rooms through multiple regularly distributed apertures. After one week of acclimatization (the 16-h LD), the plants were subjected to photoperiodic light treatments. And the daily irrigation with the multipurpose nutrient solution (macro-elements: Ca^2+^, Mg^2+^, K^+^, $NH_{4}^{+}$ , $NO_{3}^{-}$ , $SO_{4}^{2-}$ , and $H_{2}PO_{4}^{-}$ ; microelements: B, Cu, Fe, Mn, Mo, and Zn; pH = 6.5) (Yang et al., 2022) was from 8:30 ~ 9:30 a.m. Additionally, the three-replication randomized complete block design with eight plants per replication, a total of 24 plants in each treatment, and randomly located among replications to minimize the influences of light positioning in an opaque compartment.

### Light treatments

**Figure 1:**
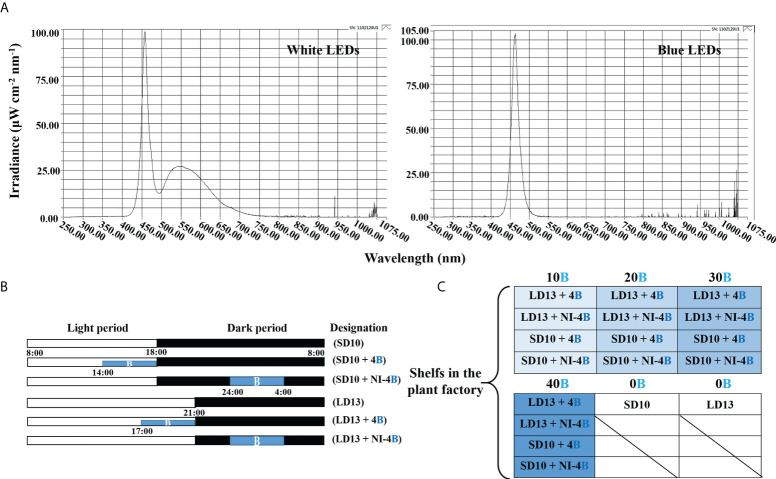
The light spectral distribution of experimental light treatments **(A)**: the daily white light (~400-720 nm, and peaked at 435 nm) provided by white LEDs and blue light (peaked at 450 nm) from blue LEDs used as the supplemental or night-interruptional light. The experimental light schemes employed in this study **(B)**: the light period started and the dark period ended at everyday 8:00 a.m.; plants in the control groups were with a 10-h short-day (SD10, positive control) or 13-h long-day (LD13, negative control) condition, without any blue light; the 4-h blue light with either 10, 20, 30, or 40 μmol·m^−2^·s^−1^ PPFD of intensities was used to (1) supplement the white light at the end of the SD10 (SD10 + 4B) and LD13 (LD13 + 4B) or (2) provide night-interruption (NI) in the SD10 (SD10 + NI-4B) and LD13 (LD13 + NI-4B). B, blue light. The experimental layout in the plant factory **(C)**: for each treatment, three replications (eight plants/replication) were located alone in an opaque compartment; the 0B, 10B, 20B, 30B, and 40B refer to the blue light with intensities of either 0, 10, 20, 30, or 40 μmol·m^−2^·s^−1^ PPFD.

The ‘Gaya Glory’ cultivar of *Chrysanthemum morifolium* shows an obligate photoperiodic flowering response in which flowering occurs under a ≥ 13-h dark period and is inhibited under a< 12-h dark period. Thus, based on our previous tests, the short-day 10 h (SD10) and long-day 13 h (LD13) photoperiods were used in this study, which can effectively initiate or inhibit flower formation, respectively (Park and Jeong, 2020). The light duration was started every day at 8:00 a.m. Plants were grown with daily light at an intensity of 300 ± 5 μmol·m^−2^·s^−1^ PPFD provided by white (W) MEF50120 LEDs (More Electronics Co. Ltd., Changwon, Korea) with a wide spectrum ranging from 400 to 720 nm and a distinct peak at 435 nm in blue (**Figure 1A**). The 4-h blue (B) (450 nm) LED light with either 0, 10, 20, 30, or 40 μmol·m^−2^·s^−1^ PPFD of intensities was used to (1) supplement the W light at the end of the SD10 (SD10 + 4B) and LD13 (LD13 + 4B) or (2) provide night-interruption (NI) in the SD10 (SD10 + NI-4B) and LD13 (LD13 + NI-4B) (**Figures 1A, B**). The control or constructed by exposing the plants to SD10 (positive control) or LD13 (negative control) conditions, without any B light. The “SD10 or LD13 + 4B (10, 20, 30, or 40)” and “SD10 or LD13 + NI-4B (10, 20, 30, or 40)” were set as experimental groups for the photoperiodic light treatments. Moreover, the experimental layout in the plant factory was shown in **Figure 1C**. Light distribution was recorded at 1 nm wavelength intervals using a spectroradiometer (USB 2000 Fiber Optic Spectrometer, Ocean Optics Inc., Dunedin, FL, USA; detects wavelength between 200 to 1000 nm) and uniformity was verified by measuring the light intensity at three points of each light treatment at the canopy level through a quantum radiation probe (FLA 623 PS, ALMEMO, Holzkirchen, Germany).

### Measurements of growth parameters

For *Chrysanthemum morifolium*, the period between the start of the SD period and flowering under optimal conditions (reaction time) can vary between 6~11 weeks (Thakur and Grewal, 2019). Based on Park and Jeong (2020), 41-days of photoperiodic B light treatment duration was enough for observing the changes in chrysanthemum photoperiodic flowering. Experimenting repeatedly, we extended the experimental duration to 60 days to ensure the complete response of chrysanthemums in each light treatment. Thus, the plant growth parameters, such as plant height, stem diameter, number of branches, leaves, and flowers per plant, and days to the first visible flower buds were collected after 60 days of light treatments. The days to visible flower buds in each treatment were determined by counting the number of days from light treatment to the date when the first flower bud appeared. The number of flowers per plant contained both blooming flowers and visible flower buds at the harvest stage. The stem diameter was measured according to the middle parts of the main stem. And the leaves with a length > 1 cm were counted to determine the total number of leaves per plant. For the biomass measurement, after careful cleaning, divided samples of shoots and roots were oven-dried (the drying oven, Venticell-222, MMM Medcenter Einrichtungen GmbH., Munich, Germany) at 85°C for five ~ seven days until a constant mass was reached to determine dry weight. Additionally, the harvested samples were kept in liquid nitrogen immediately and then stocked in the −80°C refrigerator for subsequent physiological investigations.

### Microscopic observation of stomata

After 60 days of light treatment, the stomatal traits were observed directly through fixed and discolored leaf samples due to the easiness observation of stomata in chrysanthemum leaves. At 9:00 a.m., an hour after the daily photoperiodic treatments began, the greatest stomatal opening was usually observed due to the highest photoperiodic rates. The top fourth leaves in the main stem counting from the apex (the fully expanded mature leaves) were selected from the individual plant as a biological replicate, and for each experiment, two technical and six biological replicates were performed. The excised leaf circular segments (diameter = 1 cm) were fixed at 4°C for 24 ~ 48 h in the formalin-acetic acid-alcohol (FAA) solution including 50% (*v/v*) ethanol, 45% (*v/v*) paraformaldehyde, and 5% (*v/v*) glacial acetic acid; secondly, dehydration in a graded series of ethanol solutions (95, 75, 50, 25, and 10% (*v/v*) ethanol), 15 min in each solution, three times totally; thirdly, decolorization in a mixed solution containing 45% (*v/v*) ethanol, 45% (*v/v*) acetone, and 10% (*v/v*) distilled water at 4°C for 24~48 h incubation; finally, mounted the treated sample slices on glass slides and leaf abaxial side was observed with an optical microscope (ECLIPSE Ci-L, Nikon Corporation, Tokyo, Japan) (stomatal density, magnification 20×; length and width of stomatal pores, magnification 40×), and analyzed with ImageJ (ImageJ 1.48v, NIH, USA). Additionally, the stomatal density was measured according to Sack and Buckley’s description (Sack and Buckley, 2016), while the length and width of stomatal pores were defined by Chen et al. (2012).

### Chlorophyll content

Leaf chlorophyll (Chl) content was determined according to Lichtenthaler and Buschmann (2001). After 60 days of light treatment and at 9:00 a.m., the 0.2 g of fresh leaf samples were collected from the top fourth mature leaves in the main stem counting from the apex and ground using liquid nitrogen and extracted in 2 mL 80% (*v/v*) acetone overnight at 4°C until the leaf samples were discolored completely. A UV spectrophotometer (Libra S22, Biochrom Ltd., Cambridge, UK) was used for colorimetry at A_663 nm_ and A_646 nm_. The pigment contents were calculated from the following equations: chlorophyll a (Chl a) = 12.25 × A_663_ – 2.79 × A_646_; chlorophyll b (Chl b) = 21.50 × A_646_ – 5.10 × A_663_. For each experiment, two technical and six biological replicates were performed.

### Measurement of photosynthesis and chlorophyll fluorescence

The net photosynthetic rate (*P*_n_), transpiration rate (*T*_r_), stomatal conductance (*G*_s_), and intercellular CO_2_ concentration (*C*_i_) of the top fourth fully expended mature leaves in each plant were measured by the leaf porometer (SC-1, Decagon Device Inc., Pullman, WA, USA) at the harvest time. Four positions on each leaf were measured and the average result was used. From 9:00 to 11:00 a.m., these parameters were measured in the closed-type plant factory to keep the same steady conditions.

The leaf chlorophyll fluorescence measurements were conducted using a photosystem (Fluor Pen FP 100, Photon Systems Instruments, PSI, Drásov, Czech Republic). Same as above, the top fourth fully expended mature leaves in each plant were selected for these measurements. Leaves were dark-adapted with a leaf clip for 30 min, then a 0.6 s saturating light pulse (3450 μmol·m^−2^·s^−1^ PPFD) was given to obtain the maximal fluorescence (*F*m) and minimal fluorescence (*F*0). Then, the leaf was light-adapted with 5 min continuous actinic light (300 μmol·m^−2^·s^−1^ PPFD, similar to the growing condition) with saturating pulses every 25 s, after that, the maximum light-adapted fluorescence (*F*m′) and steady-state fluorescence (*F*s) were recorded. The maximal PSII quantum yield (*F*v/*F*m) was calculated as *F*v/*F*m = (*F*m − *F*0)/*F*m (Genty et al., 1989). The actinic light was turned off and a far-red pulse was applied to obtain minimal fluorescence after the PSI excitation (*F*0′). The photochemical efficiency of PSII (*F*v′/*F*m′) was calculated as *F*v′/*F*m′ = (*F*m′ – *F*s)/*F*m′. Moreover, the photochemical quenching coefficient (*qP*) was calculated as *qP* = (*F*m′ − *F*s)/(*F*m′ − *F*0′) (Roháček, 2002). For each experiment of photosynthesis or chlorophyll fluorescence, two technical and six biological replicates were performed.

### Accumulation of carbohydrates and soluble proteins

Weighed −80°C stocked leaf samples of 0.3 g, which were harvested at 10:00 p.m. after 60 days of light treatment, and measured the contents of starch and soluble sugar based on the anthrone colorimetric method (Loewus, 1952; Yemm and Willis, 1954). For total soluble protein extraction, stocked leaf samples were collected and immediately immersed in liquid nitrogen, then ground into a fine powder over an ice bath. 0.1 g of the powder was homogenized in 50 mM PBS (1 mM EDTA, 1 mM polyvinylpyrrolidone, and 0.05% (*v*/*v*) triton-X, pH 7.0). The resulting mixture was then centrifuged (13,000 rpm, 4 ° C, 20 min) to obtain the supernatant that would be used afterward for the total protein estimation and enzyme activity assay. The total protein estimations were conducted by Bradford (1976). In addition, the contents of soluble sugars, starch, and soluble proteins were measured with a UV spectrophotometer (Libra S22, Biochrom Ltd., Cambridge, UK) at A_630 nm_, A_485 nm_, and A_590 nm_, respectively. For each experiment, two technical and six biological replicates were performed.

### Enzyme activities

The total protein solution obtained from the previous step was used to analyze the enzymatic activities and measured through a UV spectrophotometer (Libra S22, Biochrom Ltd., Cambridge, UK). For each enzymatic measurement, two technical and six biological replicates were performed. The sucrose synthase (SS) and sucrose phosphate synthase (SPS) were determined in a 1-mL reaction mixture containing a 500 μL enzyme extract at 34°C for 1 h. A 300 μL 30% (*v*/*v*) KOH was added to this mixture and was then placed in a water bath at 100°C for 10 min, after which it was gradually cooled to room temperature. The mixture was subjected to incubation at 40°C for 20 min after a 200 μL 0.15% (*v*/*v*) anthrone–sulfuric acid solution was applied and the enhancement of A_620 nm_ was monitored. The phosphoenolpyruvate carboxykinase (PEPC) was assayed in a 1 mL reaction mixture consisting of 50 mM Tris-HCl (pH 8.0), 5 mM MnCl_2_, 2 mM DTT, 10 mM NaHCO_3_, 0.2 mM NADH, 5 unit NAD-MDH, and a 160 μL enzyme extract. The reaction was initiated by adding 2.5 mM phosphoenolpyruvate (PEP). The phosphoenolpyruvate phosphatase (PEPP) was determined in a 1.5 mL reaction mixture containing 100 mM imidazole-HCl (pH 7.5), 50 mM KCl, 10 mM MgCl_2_, 0.05% (*w*/*v*) BSA, 2 mM DTT, 150 μM NADH, 1 unit LDH, 2 mM ADP, and a 150 μL enzyme extract. The reaction was initiated with 2 mM PEP, and the increase in the A_412 nm_ was monitored. The Ribulose 1,5-diphosphate carboxylase/oxygenase (RuBisCO) total activity was measured by injecting 100 μL of the supernatant into 400 μL of an assay mixture consisting of 50 mM Tris-HCl (pH 8.0), 5 mM DTT, 10 mM MgCl_2_, 0.1 mM EDTA, and 20 mM NaH_14_CO_3_ (2.0 GBq mmol^−1^) at 30°C. After a 5-min activation period, the reaction was initiated *via* the addition of RuBP to 0.5 mmol L^−1^ and was terminated after 30 s with 100 μL of 6 mol L^−1^ HCl. Moreover, the activities of soluble starch synthase (SSS), adenosine diphosphate glucose pyro-phosphorylase (ADPGPPase) and uridine diphosphate glucose pyro-phosphorylase (UDGPPase) were measured according to the protocol described by Doehlert et al. (1988) and Liang et al. (1994). The above description of enzymatic activities was conducted in accordance with the directions provided by Yang et al. (2012) and Feng et al. (2019).

### Real-time quantitative PCR verification

Total RNA was extracted using an RNeasy Plant Mini Kit (Takara Bio Inc., Tokyo, Japan), and treated with RNase-free DNase (Takara Bio Inc., Tokyo, Japan) according to the manufacturer’s instructions. A PrimeScript ^®^ Reverse Transcriptase (Takara Bio Inc., Tokyo, Japan) was used to synthesize cDNA from 1 μg of total RNA, in accordance with the manufacturer’s instructions. The cDNA was diluted 10-fold, and 5 μL was used in 15-μL quantitative RT-PCR (qRT-PCR) reactions with SYBR Premix Ex Taq™ II (Takara Bio Inc., Tokyo, Japan), performed in a Roche Light Cycler 96 real-time fluorescence quantitative PCR instrument (Roche, Basel, Switzerland). The 2^−ΔΔCt^ method (Livak and Schmittgen, 2001) was used to determine the relative expression levels of each gene. The chrysanthemum homologues of *Arabidopsis* were written as “*Cm* + *gene*” in our study. Data were averagely normalized against the expression of *CmACTIN* and *CmEF1α* (elongation factor 1α) reference genes (Gu et al., 2011; Higuchi et al., 2012). The primer sequences and PCR conditions used in the analyses were listed in **Table 1**. For each experiment, two technical and six biological replicates were performed.

**Table 1:** The primers and PCR conditions used to quantify the gene expression levels.

Name	AccessionNumber	Forward Primer(5’ to 3’)	Reverse Primer(5’ to 3’)
*CmACTIN*	AB205087	GATGACGCAGATCATGTTCG	AGCATGTGGAAGTGCATACC
*CmEF1α*	AB548817	CTTGTTGCTTGATGACTGTGG	CTTGTTGCTTGATGACTGTGG
*CmTFL1*	AB839767	CCATCATCAAGGCACAATTTCA	TTTCCCTTTGGCAGTTGAAGAA
*CDM111*	AY173054	GGTCTCAAGAATATTCGCAC	TCATTAGTCATCCCATCAGC
*CmAFL1*	AB451218	CAAGCTCAACCATCAATAGTC	TGCAGCACATGAACGAGTAG
*CmFL*	AB451217	CATTGATGCCATATTTAACTC	ACACGGATCATTCATTGTATA
*CmFTL1*	AB679270	AATCGTGTGCTATGAGAGCC	GCTTGTAACGTCCTCTTCATGC
*CmFTL2*	AB679271	ATGTGTTATTCCGGCAATTGGGTCG	AAATATGCATTTGTAACGTCATGTG
*CmFTL3*	AB679272	GGGAAAGTGGATTTGGTGGACG	GTCTTACAATTTGGTACTGTCG
*CmAFT*	AB839766	CAAGCAAAAAGCAAGGCAATCA	CAACCGGTAACCCCAAGTCATT
*CmPHYA*	AB733629	TGGAAGCAGTATGGATGCAA	TCGCAGGTATTGCACATCTC
*CmPHYB*	AB733630	TCCAAGAGGGTCATTTGGAG	ACCTGGCTAACCACAGCATC
*CmCRY1*	NM-116961	CGTAAGGGATCACCGAGTAAAG	CTTTTAGGTGGGAGTTGTGGAG
**PCR Conditions**		PCR was performed with an initial denaturing step at 95°C for 5 min, followed by 40 cycles at 95°C for 5 s, 60°C for 20 s, 72°C for 30 s, and 72°C for 10 min to final extension. Fluorescence was quantified after the incubation at 72°C.	

### Statistical analysis

In our study, all plants were randomly sampled. The data were processed, plotted, and statistically analyzed in Excel 2016 and DPS software package (DPS for Windows, 2009). Significant differences among the treatments were assessed by an analysis of variance (ANOVA), followed by Duncan’s multiple range test at a probability (*p*) ≤ 0.05 with a statistical program (SAS, Statistical Analysis System, V. 9.1, Cary, NC, USA). The differences between each treatment were tested by Student’s t-test (*p*) ≤ 0.05. Fisher’s least significant difference test was used for the *F*-test between treatments. Moreover, the experimental assays used to obtain all results were repeated six times and were presented as the mean ± standard error.

## Results

### Flowering and growth parameters

**Figure 2:**
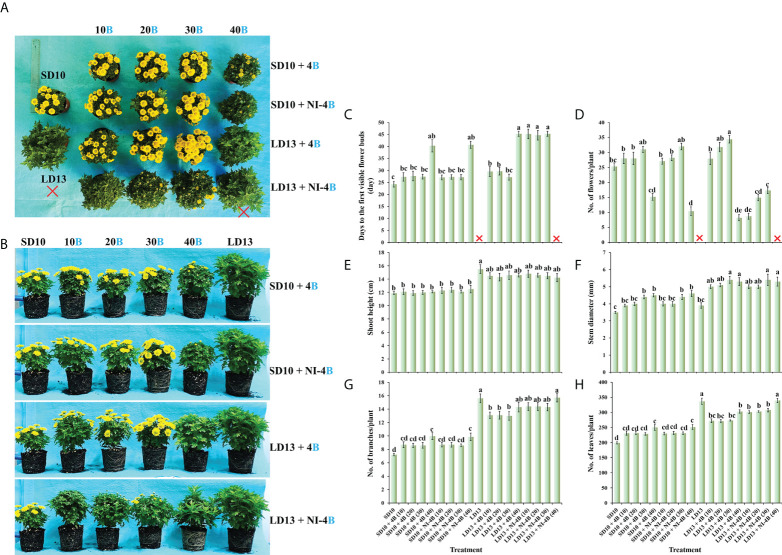
The flowering and morphology of chrysanthemum ‘Gaya Glory’ under different intensities of supplemental or night-interruptional blue light, after 60 days of exposure to the photoperiodic light treatments: the top view **(A)**, side view **(B)**, the days to the first visible flower buds **(C)**, the number of flowers per plant **(D)**, shoot height **(E)**, stem diameter **(F)**, the number of branches per plant **(G)**, and the number of leaves per plant **(H)**. The red cross, the non-flowered treatment. Vertical bars indicate the means ± standard error (n = 6). Different lowercase letters indicate significant separation within treatments by Duncan’s multiple range test at *p* ≤ 0.05. See **Figure 1** for details of photoperiodic light treatments with blue light.

In our study, the supplemental B (S-B) or night-interruptional B (NI-B) light with various intensities significantly affected the photoperiodic flowering and morphology of chrysanthemums in photoperiodic treatments. Firstly, the most interesting part is the flowering of chrysanthemums under different intensities of S-B or NI-B, after 60 days of exposure to the photoperiodic light treatments. At the harvested stage, except for LD13 (the negative control) and LD13 + NI-4B (40), these SDPs under various treatments all flowered to varying degrees (**Figure 2A**) but later than those grown under SD10 condition, especially the SD10 + 4B (40) or + NI-4B (40), LD13 + 4B (40), and LD13 + NI-4B (10, 20, or 30) treatments markedly delayed the date to the first visible flower buds (**Figure 2C**). Regardless of the photoperiodic conditions, the flowering pattern in response to the intensity of S-B or NI-B was presented consistently: gradually promoted from 10 to 30 μmol m^−2^ s^−1^ PPFD while inhibited by 40B in different degrees (**Figure 2A**). No matter the B light as the supplementary or NI in the SD conditions, it did no significant difference in the total flower number per plant with the same light intensity. While in LD conditions, when compared with the NI-B, the S-B with any intensity obviously increased the total number of blooming flowers and flower buds, especially the LD13 + 4B (30) led to the most flower number, however, the LD13 + NI-4B (10, 20, or 30) notably decreased flowers, until LD13 + NI-4B (40) completely inhibited flowering in chrysanthemums (**Figure 2D**).

Secondly, we also investigated how the morphology of chrysanthemums in response to the intensity of S-B or NI-B in photoperiodic light treatments. As shown in **Figures 2B, E**, the plants grown in LD conditions are usually higher than those in SD environments, especially the LD13 caused the highest plants during all treatments. In LDs, applied various intensities of S-B or NI-B non-differently and slightly shortened the shoot height than LD13. However, there were no significant differences in shoot height among all plants which were grown under SD conditions. Moreover, our results showed that the B light benefitted in improving the stem diameter, especially in the LD conditions, the thickest stems were observed in 30 or 40B, regardless of the S-B or NI-B (**Figure 2F**). Additionally, the LD conditions appeared to be more favorable to the formation of leaves and branches than SDs, and the non-flowered treatments of LD13 and LD13 + NI-4B (40) resulted in the most branches and leaves among all treatments. Moreover, no matter the B light as the supplementary or NI in photoperiodic treatments, similar changing patterns in the number of leaves and branches were observed under different blue light intensities: more flowers and fewer branches or leaves (**Figures 2G, H**), which was shown a kind of competitive relationship between flower induction with leaf or branch formation.

### Stomata characteristics

**Figure 3:**
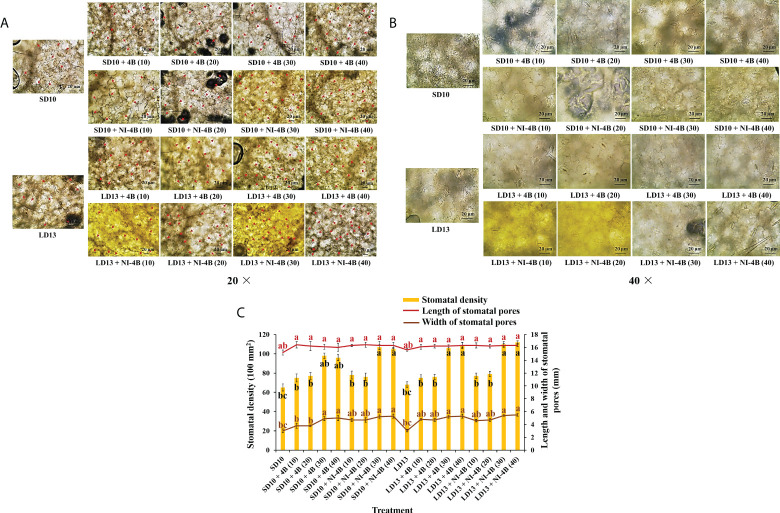
The stomatal traits of chrysanthemum ‘Gaya Glory’ under different intensities of supplemental or night-interruptional blue light, after 60 days of exposure to the photoperiodic light treatments: the micrographs of stomatal density (magnification 20×) **(A)** and specific state of stomatal pores (magnification 40×) **(B)**; the analysis chart **(C)** of stomatal density and pores. The red arrows indicate the locations of stomata in Figure **(A)** Bars indicate 20 μm in Figures A and **(B)** Vertical bars indicate the means ± standard error (n = 6). Different lowercase letters indicate significant separation within treatments by Duncan’s multiple range test at *p* ≤ 0.05. See **Figure 1** for details of photoperiodic light treatments with blue light.

**Figure 3** shows the effects of S-B or NI-B intensity on the stomatal traits of chrysanthemums in photoperiodic treatments, and a notable interaction was observed for the different stomatal parameters. SD10 + NI-4B (30 or 40), LD13 + 4B or + NI-4B (30 or 40) significantly increased the stomatal density, followed by SD10 + 4B (30 or 40), however, it did not differently respond to the 0, 10, or 20 μmol m^−2^ s^−1^ PPFD of S-B or NI-B in both LDs and SDs (**Figures 3A, C**). Moreover, the stomatal aperture parameters were also influenced by different light treatments but mainly responded to the B light intensity. SD10 and LD13 led to the minimum stomatal aperture width and length, and then the second smallest aperture width was observed in SD10 + 4B (10 or 20), other treatments all obviously promoted stomatal opening, especially the 30 and 40B which were caused the greatest stomatal aperture width. There were no significant effects of S-B or NI-B intensity and photoperiods on stomatal aperture length (**Figures 3B, C**).

### Chlorophyll content

**Figure 4:**
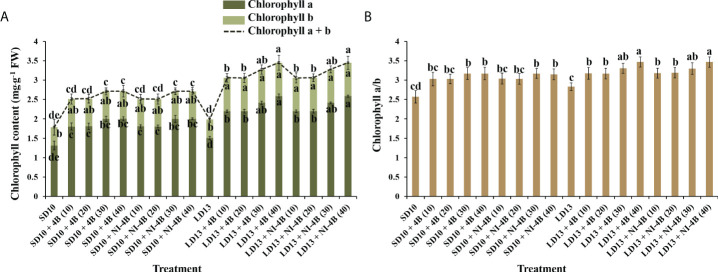
The effects on the chlorophyll content of chrysanthemum ‘Gaya Glory’ under different intensities of supplemental or night-interruptional blue light, after 60 days of exposure to the photoperiodic light treatments: the contents of chlorophyll a, b, and a + b **(A)** and chlorophyll a/b **(B)**. Vertical bars indicate the means ± standard error (n = 6). Different lowercase letters indicate significant separation within treatments by Duncan’s multiple range test at *p* ≤ 0.05. See **Figure 1** for details of photoperiodic light treatments with blue light.

Different intensities of S-B or NI-B in photoperiodic treatments significantly affected the leaf Chl content and the value of Chl a/b. From our results in **Figure 4A**, LD conditions promoted more Chl accumulation than SDs. Chl a was more sensitive than Chl b in response to the S-B or NI-B intensity, and the 30 and 40B usually caused the most Chl a, followed by 10 and 20B, while 0 μmol m^−2^ s^−1^ PPFD of B light resulted in the minimum content of Chl a. The content of Chl b was comparatively stable under photoperiodic treatments and showed non-difference within 10, 20, 30, and 40B but always higher than 0B, though this promotion was slightly obvious in LD conditions. Thus, the changing pattern of Chl a + b was the same as Chl a. Similar to the changing trend of Chl a content, the Chl a/b ratios also increased by 30 and 40B, and then were 10 and 20B, however, the overall change tends to be more moderate and with little difference (**Figure 4B**).

### Photosynthetic index and chlorophyll fluorescence

In photoperiodic treatments, the various intensities of S-B or NI-B signally affected the photoperiodic indexes, such as *P*n, *T*r, *G*s, and *C*i (**Table 2**). The 30 and 40B interacted with the NI in LDs significantly increased the *P*n, followed by LD13 + 4B (30 or 40), and LD13 + 4B or + NI-4B (10 or 20) did no difference in *P*n promotion, but always better than 0B. The changing pattern of *P*n in SDs was shown that SD10 + 4B and + NI-4B (30 or 40) ≥ SD10 + 4B and + NI-4B (10 or 20) > SD10. And this promotion on *P*n was more obvious in LD conditions. Moreover, other photosynthetic indexes (*T*r, *G*s, and *C*i) were highly related to the stomatal state and mainly responded to the B light intensity. Consistent with the changing patterns of stomatal density and aperture width (**Figure 3C**), the 30 and 40B of supplementary or NI always induced the greatest values of *T*r, *G*s, and *C*i, and then were 10 and 20B, 0B always resulted in the lowest values of those, regardless of the photoperiods.

**Table 2:** The photosynthetic and chlorophyll fluorescence characteristics of chrysanthemum ‘Gaya Glory’ under different intensities of supplemental or night-interruptional blue light, after 60 days of exposure to the photoperiodic light treatments.

Photoperiod (I)	Blue light treatment (II)	Blue light intensity (III)	*P*n^1^(μmol CO_2_ m^-2.^s^-1^)	*T*r^2^(mmol H_2_O m^-2.^s^-1^)	*G*s^3^(mol H_2_O m^-2.^s^-1^)	*C*i^4^(μmol CO_2_ mol^-1^)	*F*v/*F*m^5^	*F*v′/*F*m′^6^	*qP* ^7^
SD10	None	0	12.98 ± 0.12e^8^	1.49 ± 0.02c	0.38 ± 0.012c	323.23 ± 2.12b	0.77 ± 0.011bc	0.41 ± 0.008c	0.39 ± 0.002cd
	+ 4B	10	16.54 ± 0.34d	1.79 ± 0.04b	0.58 ± 0.010b	412.17 ± 3.14ab	0.83 ± 0.017ab	0.56 ± 0.003ab	0.51 ± 0.003b
		20	16.72 ± 0.27d	1.80 ± 0.03b	0.60 ± 0.013b	413.03 ± 1.56ab	0.83 ± 0.009ab	0.55 ± 0.007ab	0.53 ± 0.004b
		30	18.43 ± 0.36cd	2.09 ± 0.01ab	0.79 ± 0.009a	472.14 ± 3.79a	0.87 ± 0.013a	0.59 ± 0.010a	0.55 ± 0.004ab
		40	18.67 ± 0.41cd	2.10 ± 0.03ab	0.81 ± 0.014a	469.99 ± 4.23a	0.88 ± 0.017a	0.58 ± 0.009a	0.56 ± 0.011ab
	+ NI-4B	10	16.31 ± 0.23d	1.80 ± 0.04b	0.60 ± 0.010b	411.16 ± 2.31ab	0.83 ± 0.010ab	0.56 ± 0.014ab	0.52 ± 0.010b
		20	16.27 ± 0.20d	1.78 ± 0.02b	0.61 ± 0.009b	413.01 ± 2.47ab	0.84 ± 0.024ab	0.56 ± 0.011ab	0.52 ± 0.009b
		30	18.79 ± 0.34cd	2.11 ± 0.01ab	0.80 ± 0.007a	473.56 ± 3.01a	0.87 ± 0.012a	0.59 ± 0.008a	0.56 ± 0.005ab
		40	18.83 ± 0.47cd	2.10 ± 0.02ab	0.80 ± 0.014a	479.72 ± 5.23a	0.88 ± 0.015a	0.59 ± 0.005a	0.56 ± 0.010ab
LD13	None	0	19.98 ± 0.56c	1.50 ± 0.01c	0.40 ± 0.011c	367.38 ± 4.38b	0.80 ± 0.014b	0.50 ± 0.004b	0.43 ± 0.004c
	+ 4B	10	21.76 ± 0.60b	1.81 ± 0.03b	0.63 ± 0.007b	411.11 ± 3.79ab	0.84 ± 0.011ab	0.57 ± 0.011ab	0.55 ± 0.007ab
		20	21.51 ± 0.34b	1.80 ± 0.02b	0.63 ± 0.012b	412.09 ± 1.20ab	0.85 ± 0.012ab	0.56 ± 0.009ab	0.55 ± 0.003ab
		30	23.98 ± 0.62ab	2.26 ± 0.04a	0.82 ± 0.010a	473.17 ± 2.00a	0.87 ± 0.013a	0.60 ± 0.003a	0.57 ± 0.006a
		40	24.03 ± 0.11ab	2.27 ± 0.03a	0.81 ± 0.011a	475.03 ± 4.12a	0.87 ± 0.011a	0.59 ± 0.005a	0.58 ± 0.005a
	+ NI-4B	10	21.53 ± 0.24b	1.80 ± 0.01b	0.64 ± 0.008b	410.02 ± 4.07ab	0.85 ± 0.015ab	0.56 ± 0.003ab	0.55 ± 0.007ab
		20	21.64 ± 0.31b	1.79 ± 0.01b	0.65 ± 0.005b	410.17 ± 3.17ab	0.84 ± 0.007ab	0.55 ± 0.004ab	0.56 ± 0.009ab
		30	24.99 ± 0.29a	2.30 ± 0.02a	0.82 ± 0.012a	478.23 ± 4.23a	0.88 ± 0.009a	0.60 ± 0.007a	0.60 ± 0.006a
		40	25.01 ± 0.17a	2.29 ± 0.03a	0.83 ± 0.007a	479.07 ± 5.37a	0.88 ± 0.005a	0.61 ± 0.001a	0.59 ± 0.011a
*F*-test	I		***	NS	NS	NS	*	**	**
	II		***	***	***	**	**	**	***
	III		**	***	***	**	**	**	**
	I × II		***	**	**	**	**	**	**
	I × III		***	**	**	*	*	*	**
	II × III		***	***	***	***	**	**	**
	I × II × III		***	***	***	***	***	**	***

^1.^ Net photosynthetic rate (*P*n). ^2.^ Transpiration rate (*T*r). ^3.^ Stomatal conductance (*G*s). ^4.^ Intercellular CO_2_ concentration (*C*i). ^5.^ The maximal PSII quantum yield (*F*v/*F*m). ^6.^ The photochemical efficiency of PSII (*F*v′/*F*m′). ^7.^ The photochemical quenching coefficient (*qP*). ^8.^ Mean separation within columns by Duncan’s multiple range test at *p* ≤ 0.05 and the values are average ± standard error (n = 6). NS, *, **, and *** mean non-significant or significant at *p* ≤ 0.05, 0.01, or 0.001, respectively. See **Figure 1** for details of photoperiodic light treatments with blue light.

Furthermore, the intensity of S-B or NI-B in photoperiodic treatments also influenced chlorophyll fluorescence parameters in chrysanthemums (**Table 2**). A similar trend of *F*v/*F*m, *F*v’/*F*m’, and *qP* was observed, from highest to lowest: LD13 + NI-4B (30 or 40) = LD13 + 4B (30 or 40) = SD10 + NI-4B (30 or 40) = SD10 + 4B (30 or 40) ≥ LD13 + NI-4B (10 or 20) = LD13 + 4B (10 or 20) ≥ SD10 + NI-4B (10 or 20) = SD10 + 4B (10 or 20) > LD13 ≥ SD10. Our results showed that applying 30 or 40 μmol m^−2^ s^−1^ PPFD of B light, especially in LD conditions significantly improved the light energy conversion efficiency of the PSII reaction center and enhanced the actual light energy capture efficiency. Eventually, LD13 + 4B or + NI-4B (30 or 40) led to the greater *P*n among all treatments.

### Plant dry mass, accumulation of carbohydrates and soluble proteins

**Figure 5:**
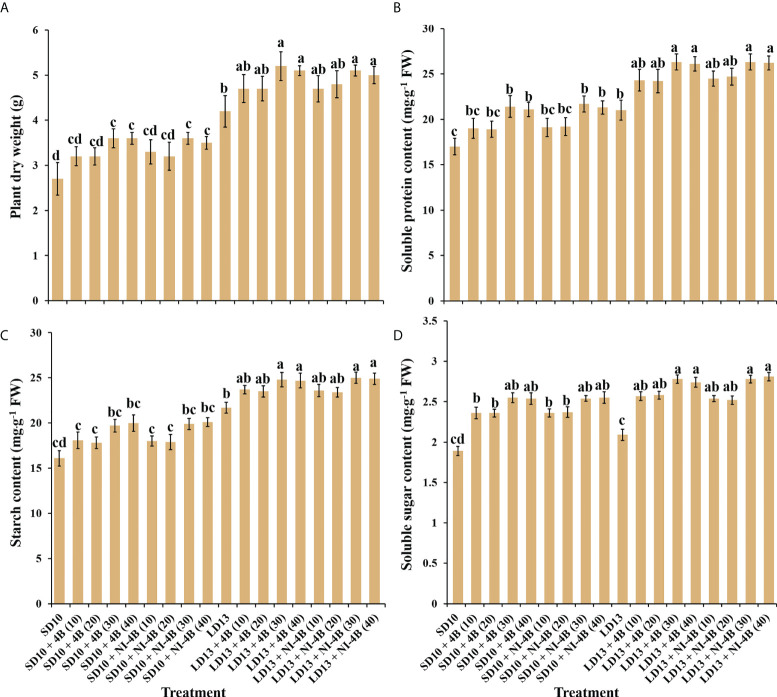
The effects on the plant dry weight **(A)**, soluble protein **(B)**, starch **(C)**, and soluble sugar **(D)** contents of chrysanthemum ‘Gaya Glory’ under different intensities of supplemental or night-interruptional blue light, after 60 days of exposure to the photoperiodic light treatments. Vertical bars indicate the means ± standard error (n = 6). Different lowercase letters indicate significant separation within treatments by Duncan’s multiple range test at *p* ≤ 0.05. See **Figure 1** for details of photoperiodic light treatments with blue light.

The plant dry mass, accumulation of carbohydrates and soluble proteins differently responded to the intensity of S-B or NI-B in photoperiodic treatments (**Figure 5**). The chrysanthemums under LD conditions generally represented the obvious higher plant dry mass and contents of starch and soluble proteins than SDs, while the changing pattern of soluble sugar content in response to the photoperiods was not significant. Furthermore, the intensity of S-B or NI-B in photoperiodic treatments differently increased the accumulation of organic nutrients, 40B and 30B performed the best, followed by 20B and 10B, and always better than 0B, regardless of the photoperiods.

### Enzyme activities

**Figure 6:**
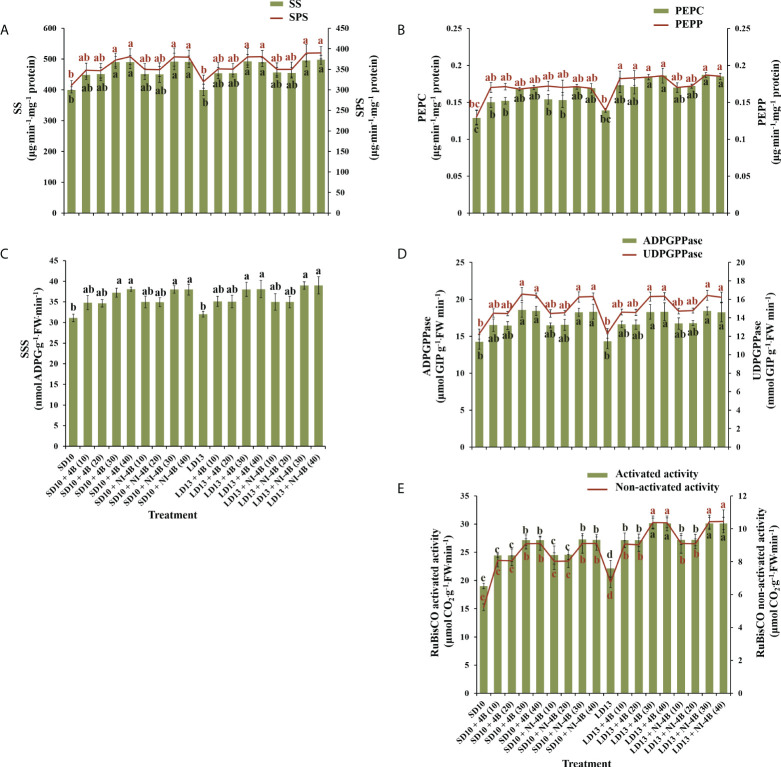
The effects on the enzymatic activities of chrysanthemum ‘Gaya Glory’ under different intensities of supplemental or night-interruptional blue light, after 60 days of exposure to the photoperiodic light treatments. Sucrose synthesis enzymes: **(A)** sucrose synthase (SS) and sucrose phosphate synthase (SPS), **(B)** phosphoenolpyruvate carboxykinase (PEPC) and phosphoenolpyruvate phosphatase (PEPP). Starch synthesis enzymes: **(C)** soluble starch synthase (SSS), **(D)** adenosine diphosphate glucose pyro-phosphorylase (ADPGPPase) and uridine diphosphate glucose pyro-phosphorylase (UDGPPase). And photosynthesis-related enzyme: **(E)** the activated or non-activated activity of Ribulose 1,5-diphosphate carboxylase/oxygenase (RuBisCO). Vertical bars indicate the means ± standard error (n = 6). Different lowercase letters indicate significant separation within treatments by Duncan’s multiple range test at *p* ≤ 0.05. See **Figure 1** for details of photoperiodic light treatments with blue light.

We further investigated the activities of carbohydrate synthesis and photosynthesis-related enzymes in chrysanthemums to explore the response to various intensities of S-B or NI-B in photoperiodic treatments (**Figure 6**). In general, regardless of the photoperiods, S-B, or NI-B, the sucrose synthesis-related enzymes (sucrose synthase (SS), sucrose phosphate synthase (SPS), phosphoenolpyruvate carboxykinase (PEPC), and phosphoenolpyruvate phosphatase (PEPP)) and starch synthesis-related enzymes (soluble starch synthase (SSS), adenosine diphosphate glucose pyro-phosphorylase (ADPGPPase), and uridine diphosphate glucose pyro-phosphorylase (UDGPPase)) were mainly responded to the intensity, still, the 30B and 40B were the best-performing intensities while the promotion in enzymatic activity of 10B and 20B were slightly weak (**Figures 6A-D**). A similar response of total activities (both activated and non-activated) of Ribulose 1,5-diphosphate carboxylase/oxygenase (RuBisCO) to B light intensity was performed as above, while the promotion of 30B and 40B was more obvious in LD conditions (**Figure 6E**).

### Expression level of photoreceptors and flowering-related genes

**Figure 7:**
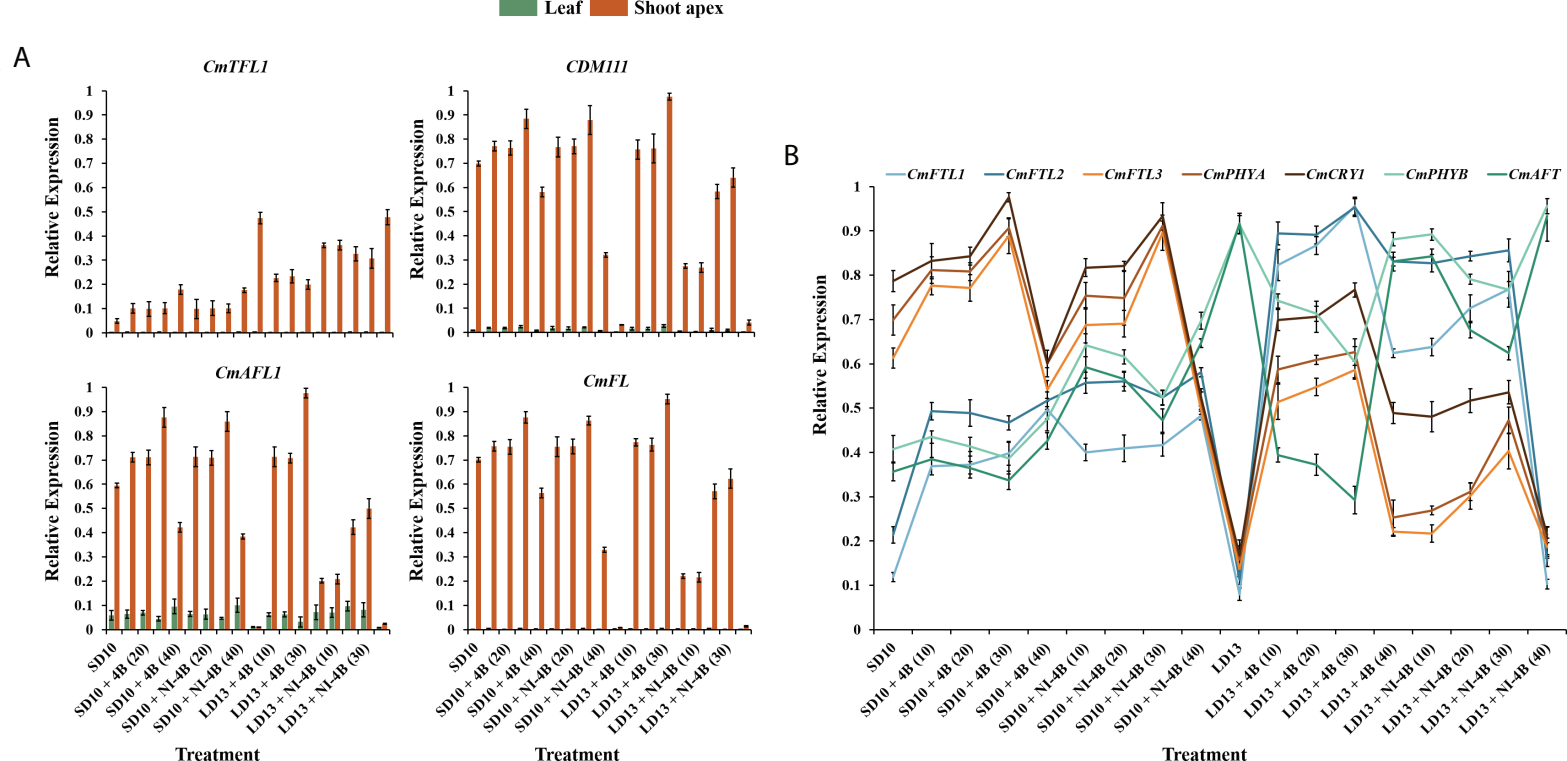
Expression patterns of flowering-related genes in chrysanthemum ‘Gaya Glory’ under different intensities of supplemental or night-interruptional blue light, after 60 days of exposure to the photoperiodic light treatments: **(A)** the tissue-specific expression patterns of flowering-related genes in leaves and shoot apexes and **(B)** the expression levels of flowering or photoreceptor-related genes in leaves. The top leaves (the fourth true leaves from the shoot apex) and shoot apexes were harvested at ZT4 (4 h after lights-on, from 8:00 a.m.) for RNA extraction and RT-PCR. Data were averagely normalized against the expression of *CmACTIN and CmEF1α*. The maximum value in each experiment was set to “1”. Vertical bars indicate the means ± standard error of six biological replicates (n = 6), using RNA from separate plants. See **Figure 1** for details of photoperiodic light treatments with blue light.

To study the tissue-specific expression patterns of flowering-related genes in *C. morifolium*, the chrysanthemum homologues of *Arabidopsis*: the anti-florigenic *TFL1/CEN*-like gene (*CmTFL1*) (Higuchi et al., 2013), and three well-characterized floral meristem identity genes *APETALA1* (*CDM111*), *FRUITFULL* (*CmAFL1*), and *LEAFY* (*CmFL*) (Shchennikova et al., 2004; Li et al., 2009) were selected and analyzed by qRT-PCR in leaves and shoot apexes, respectively (**Figure 7A**). After 60 days of exposure to the photoperiodic light treatments, at the harvest stage, these floral forming-related genes were all highly expressed in shoot apices, in contrast, the extremely lower or barely detectable expression was observed in leaves. The expression of the anti-florigenic gene *CmTFL1* was generally higher in LD conditions than in SDs. And highly expressed in treatments with significantly inhibited flowering or no flowering, especially in LD13 and LD13 + NI-4B (40) treatments. The general rule of this anti-florigenic gene was: inversely proportional to flowering capacity. The tissue-specific expression patterns of three floral meristem identity genes *CDM111*, *CmAFL1*, and *CmFL* were roughly the opposite of *CmTFL1*, highly expressed in SD10 + 4B, SD10 + NI-4B, and LD13 + 4B treatments, and gradually promoted from 10 to 30 μmol m^−2^ s^−1^ PPFD and inhibited by 40B. Moreover, they were generally lower in LD13 + NI-4B and barely expressed in non-flowered LD13 and LD13 + NI-4B (40) treatments. The general rule of these floral meristem identity genes was: proportional to flowering capacity. Overall, these expression levels were correlated with the extent of flower induction in our study (**Figure 2A**).

The expression pattern of photoreceptor or flowering-related homologues of *Arabidopsis* in *Chrysanthemum morifolium* leaves after 60 days of photoperiodic light treatments were also investigated (**Figure 7B**). The *FT*-like genes (*CmFTL1*, *CmFTL2*, and *CmFTL3*) (Higuchi et al., 2012; Sun et al., 2017), anti-florigenic FT/TFL1 family *TFL1/CEN/BFT*-like gene (*CmAFT*) (Zhao et al., 2022), and three photoreceptor genes [*Phytochrome A* (*CmPHYA*), *Phytochrome B* (*CmPHYB*), and *Cryptochrome 1* (*CmCRY1*)] (Higuchi et al., 2012; Park et al., 2015) were selected. Their expression patterns can be broadly classified into three types: (1) one florigen gene *CmFTL3* and two photoreceptor genes—*CmPHYA* and *CmCRY1* in *Chrysanthemum morifolium* highly expressed in SD10 + 4B, SD10 + NI-4B, and LD13 + 4B treatments and gradually promoted from 10B to 30B and inhibited by 40B. Moreover, they were generally lower in LD13 + NI-4B and barely expressed in non-flowered LD13 and LD13 + NI-4B (40) treatments; (2) the anti-florigenic gene *CmAFT* and a photoreceptor gene *CmPHYB* were expressed like the expression pattern of *CmTFL1* in **Figure 7A**, generally higher in LD conditions and highly expressed in treatments with significantly inhibited flowering or no flowering, especially in LD13 and LD13 + NI-4B (40) treatments; (3) The *CmFTL1* and *CmFTL2* were highly expressed in the leaves under flower inductive conditions (LD13 + 4B (10, 20, 30, or 40) and LD13 + NI-4B (10, 20, or 30)), but relatively lower in SD conditions and really poor in non-flowered LD13 and LD13 + NI-4B (40) treatments. The constitutive expression of *CmFTL1* and *CmFTL2* in *Chrysanthemum morifolium* leaves revealed weak florigenic activity. *CmFTL1* and *CmFTL2* might function as an LD florigen similar to *RICE FLOWERING LOCUS T1* (*RFT1*) as suggested in rice, a facultative SDP (Komiya et al., 2009).

**Figure 8:**
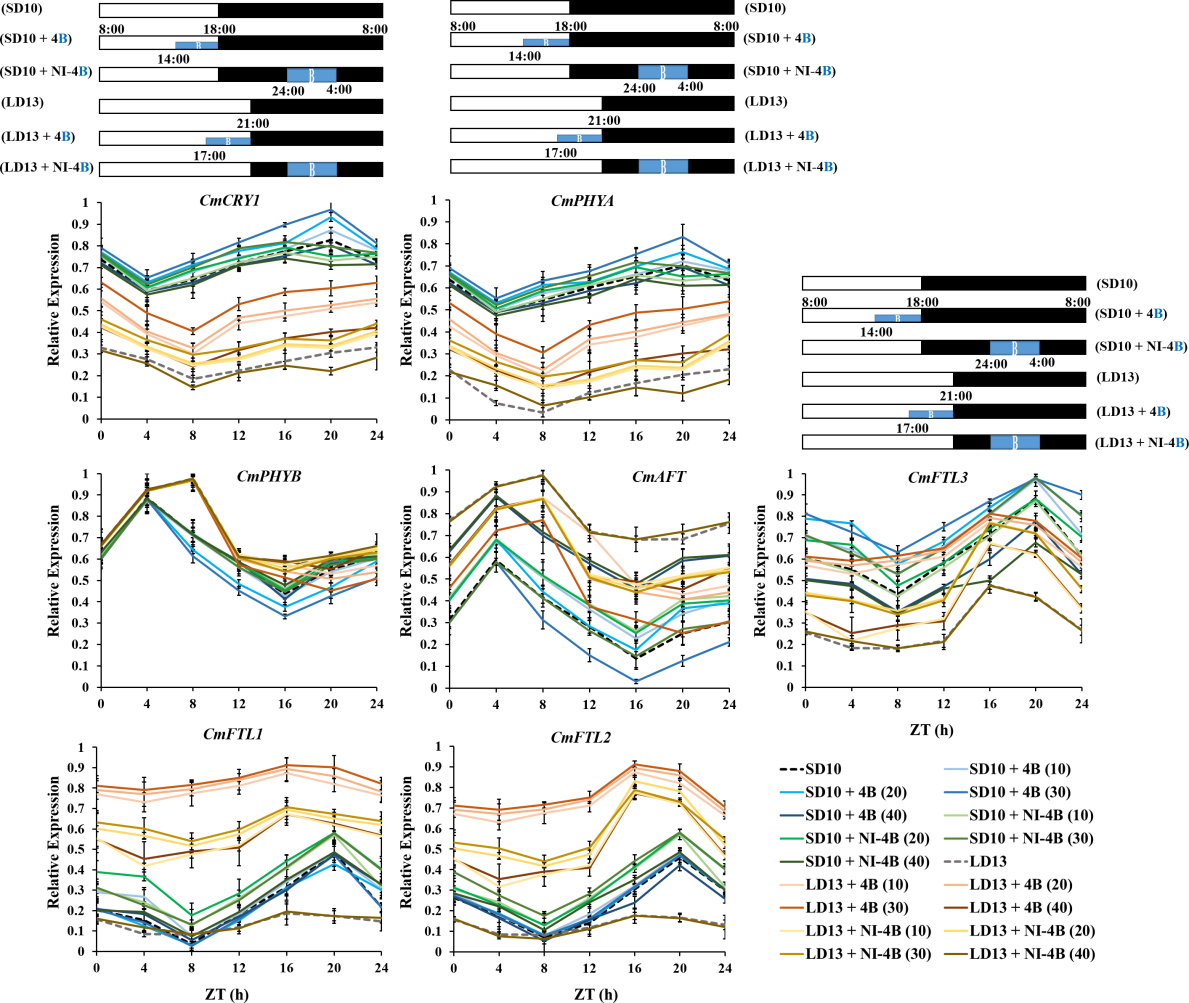
The temporal expression patterns of flowering-related genes in leaves of chrysanthemum ‘Gaya Glory’ under different intensities of supplemental or night-interruptional blue light, after 7 days of exposure to the photoperiodic light treatments. For RNA extraction and RT-PCR, the top leaves (the fourth true leaves from the shoot apex) were harvested at 0, 4, 8, 12, 16, 20, and 24h after lights-on (from 8:00 a.m.), respectively (ZT 0, 4, 8, 12, 16, 20, and 24). The horizontal white and black bars represent the period of day and night, respectively; the blue bars represent the periods with different intensities of supplemental or night-interruptional blue light. Data were averagely normalized against the expression of *CmACTIN and CmEF1α*. The maximum value in each experiment was set to “1”. Vertical bars indicate the means ± standard error of six biological replicates (n = 6), using RNA from separate plants. See **Figure 1** for details of photoperiodic light treatments with blue light.

Because the photoreceptors and flowering-related genes in chrysanthemum have a clear diurnal rhythm and they fluctuate a lot depending on the light or dark conditions. Thus we also studied the temporal expression patterns of *CmFTL1*, *CmFTL2*, *CmFTL3*, *CmAFT*, *CmPHYA*, *CmPHYB*, and *CmCRY1* in leaves of *Chrysanthemum morifolium* (**Figure 8**). After 7 days of exposure to the photoperiodic light treatments, the top leaves were harvested at 0, 4, 8, 12, 16, 20, and 24h after lights-on (from 8:00 a.m.), respectively. Based on the pattern of curve changes within 24 hours, we roughly divided these seven genes into three groups: (1) one florigen gene *CmFTL3* and two photoreceptor genes—*CmPHYA* and *CmCRY1*; (2) the anti-florigenic gene *CmAFT* and a photoreceptor gene *CmPHYB*; (3) the LD florigen*-RFT1* like genes *CmFTL1* and *CmFTL2*.

Firstly, the expression patterns of *CmFTL3*, *CmPHYA*, and *CmCRY1* expressed similarly *and* showed clear diurnal rhythms, which dropped to a low point at the beginning of the lights-on periods (4 h or 8 h after lights-on in SD and LD conditions), and peaked at the beginning of the NI-B (16 h after lights-on in LDs) or at the end of the NI-B (20 h after lights-on in SDs). Moreover, the expression of those three genes was highest under SD conditions, lowest under LD13 and LD13 + NI-4B conditions, and intermediate under LD13 + 4B conditions. Secondly, the expression patterns of *CmAFT* and *CmPHYB* peaked at the beginning of the S-B (4 h or 8 h after lights-on in SD or LD conditions, respectively) and dropped to a low point at the beginning of the NI-B (16 h after lights-on in SDs) or at the end of the NI-B (20 h after lights-on in LDs). Both *CmAFT* and *CmPHYB* were highly expressed in LD conditions. The expression of *CmAFT* was scattered between treatments, and the highest expression levels were observed in non-flowered treatments LD13 and LD13 + NI-4B (40). Thirdly, the expression patterns of *CmFTL1* and *CmFTL2* were similar, the expression was higher under the flower-inducted treatments in LD conditions, lower under non-flowered LD13 and LD13 + NI-4B conditions, and intermediate under SD conditions. And they peaked at the beginning of the NI-B (16 h after lights-on in LDs) or at the end of the NI-B (20 h after lights-on in SDs). Furthermore, the expression of both *CmFTL3* and *CmAFT* was regulated by the light-signalling-mediated gene *CmPHYB*, up-regulated *CmAFT*, and down-regulated *CmFTL3*, which also strengthened our confidence in photoperiodic flowering regulated by photoreceptor-mediated control.

## Discussion

### The growth and physiology of chrysanthemum plant in response to intensity of supplemental or night-interruptional blue light in photoperiodic treatments

Light affects plant growth and development by lighting duration (photoperiod), intensity, and quality (Kami et al., 2010). Within the appropriate range of lighting intensity, the higher daily light integral (DLI) plants receive, the stronger they grow and produce a higher yield. Thus, for greater vegetative growth it is usually better to provide the optimal light intensities over a longer photoperiod (Pearcy, 2000). In SD periods, the non-produced night time is longer than the light duration, and plants tend to spare accumulated sugars by limiting their growth (Gent, 2018). In our current study, compared to SD environments, all LD conditions generally improved plant height (**Figure 2E**), stem diameter (**Figure 2F**), total chlorophyll content (**Figure 4A**), plant dry weight (**Figure 5A**), soluble protein content (**Figure 5B**), and starch content (Figure 5C). The photoperiod is not only involved in energy provision but also in the regulation of branch or leaf formation. SDPs only grow nutritionally and do not flower in LD conditions (Garner, 1933). Consistent with our results that the LDs caused more branches and leaves in SDP chrysanthemum (**Figures 2G, H**). Moreover, a higher net photosynthetic rate (*P*n) was observed in LD conditions (**Table 2**), which was due to the continued respiration during the day and night.

Furthermore, the B light effect in plants involves various aspects, such as photoreceptors, signal transduction, pigment biosynthesis, carbon metabolism, nitrogen metabolism, chloroplast development, morphogenesis, and stomatal movement et al. The shortest plants, thickest stems, the best protein content, carbohydrate content, and dry matter quality are usually observed under B light than in other conditions (Xu et al., 2015). In general, B light is required for chlorophyll synthesis, chloroplast formation, high chlorophyll a/b ratio, and high photosynthetic rates in higher plants (Thomas, 1981). B light promotes the *de novo* synthesis of the core protein D1 (QB) protein of the PSII reaction center complex (Richter and Wessel, 1985), thereby promoting photosynthesis. Stomatal conductance, transcript levels of key photosynthetic genes, total soluble sugars and sucrose, and starch content are higher in plants grown under B light than those grown under white light (Wang et al., 2009). Moreover, B light not only activates many enzymes in the carbohydrate synthesis, photosynthetic carbon assimilation and photorespiration, and chlorophyll synthesis pathways, but also induces the synthesis of Rubisco (Hundrieser and Richter, 1982; Roscher and Zetsche, 1986), uridine diphosphate glucose (UDPG) pyro-phosphorylase, and PEPC (Kamiya and Miyachi, 1975) to enhance Rubisco and nicotinamide adenine dinucleotide phosphate (NADP)-dependent phosphoglycerate dehydrogenase (Conradt and Ruyters, 1980). Supported by those positive effects of B light in plant growth and development, S-B and NI-B applied in photoperiodic light treatment differently improved those morphological and physiological traits which are mentioned above, and generally, the 30 and 40 μmol m^−2^ s^−1^ PPFD of S-B or NI-B were more effective in improving chrysanthemum growth and development regardless of the photoperiod. However, the intensity of S-B or NI-B during photoperiodic treatments did non-significant effects on some growth or physiological indexes, such as the shoot height (**Figure 2E**), stomatal aperture length (**Figure 3C**), and chlorophyll b content (**Figure 4A**), which might due to reason of the B light accounting for the proportion of the main white light is too weak. Overall, various intensities of S-B or NI-B applied in photoperiodic light treatments effectively promote the chrysanthemum growth and physiology, while the B light does not work completely alone and interacted with the photoperiod to co-regulate the plant development.

### The flowering and branching of chrysanthemum plant in response to the *CmTFL1* under various intensities of supplemental or night-interruptional blue light in photoperiodic treatments

According to Gao et al. (2019), the secondary branching in *Arabidopsis* and the axillary buds in *Chrysanthemum* were all improved by the *CmTFL1* gene, which indicated that the high expression level of *CmTFL1* in stem promoted the development of lateral meristems. Similar phenomena were seen in other species with homologous *TFL1* genes. In *Lolium perenne* L., the *LpTFL1* gene not only recovered the *tfl1* mutant’s phenotype but also produced a high number of secondary branches and leaves with better vegetative development (Jensen et al., 2001). Moreover, the *AtTFL1* in *Arabidopsis*, the *PsTFL1* in *Prunus serotine*, and the *LjCEN1* gene in *Lotus japonicas* all increased the number of branches and leaves (Ratcliffe et al., 1998; Guo et al., 2006; Wang and Pijut, 2013). Therefore, the *TFL1* gene shows a conserved function in regulating branching and leafing. Additionally, the constitutive expression of *CsTFL1* was extremely delayed in the flower formation in SD conditions of *Chrysanthemum seticuspe*. Further verified the function of *CmTFL1* with five transgenic lines, and showed that *CmTFL1* functionally affected flower development in *Chrysanthemum morifolium* (Higuchi et al., 2013; Higuchi and Hisamatsu, 2015). Similarly, transgenic *JcTFL1b*-RNAi *Jatropha* consistently presented a relatively early flowering (Li et al., 2017). In our current study, no matter the S-B or NI-B, similar changing patterns in the number of leaves and branches were observed under different blue light intensities: more branches or leaves and fewer flowers (**Figures 2G, H**). And the expression of *CmTFL1* was generally higher in LD conditions, and highly expressed in treatments with an obvious more number of branches and leaves, especially in LD13 and LD13 + NI-4B (40) treatments (**Figure 7A**). Overall, the *CmTFL1* gene positively regulates branching and leafing but inhibits flowering, which makes the plant bushier. Therefore, *CmTFL1* can as a candidate gene for regulating both the production and quality of plants.

### Chrysanthemum flowering in response to photoreceptor-mediated florigenic and anti-florigenic genes under various intensities of supplemental or night-interruptional blue light in photoperiodic treatments

It is commonly established that inductive photoperiods induce leaves to synthesize a floral stimulus (“florigen”). It has been postulated, nevertheless, that an anti-florigenic signal generated in leaves may control photoperiodic floral induction; the correct day duration would then result in the elimination of an anti-florigen. (Lang and Melchers, 1943; Thomas and Vince-Prue, 1996). AFT, an anti-florigenic FT/TFL1 family protein, was discovered in *Chrysanthemum seticuspe*, and it was convincingly demonstrated that CsAFT protein operates as a systemic floral inhibitor—an anti-florigenic signal generated in leaves under non-inductive circumstances. And the investigation of photoperiodic responses of *CsAFT*-RNAi plants supports the need for the anti-florigenic signal (CsAFT) to sustain the vegetative state (Higuchi et al., 2013). Thus, it is most important for active flowering through the photoperiodic regulation in florigen synthesis, and the *CmPHYB*-mediated anti-florigen *CmAFT* gene also has a predominant role in the obligatory photoperiodic flowering response in chrysanthemum, enabling for strict vegetative maintenance under non-inductive photoperiods (**Figures 2A**, **7B**, and **8**). Chrysanthemum is an obligate SDP that remains vegetative in the absence of inductive LD conditions, such as LD13 and LD13 + NI-4B (40) treatments in our study (**Figure 2A**). Nevertheless, rice (*Oryza sativa*), a facultative SDP, may blossom even in non-inductive LD circumstances. Two florigen genes (*Hd3a* and *RFT1*) of rice are activated depending on the length of the day, and the RFT1 protein is proposed as the LD florigen (Komiya et al., 2009). In the chrysanthemum, *CmFTL1* might similar to *RFT1* in rice and function as an LD florigen gene. It can be assumed that residual *CmFTL3* and increased *CmFTL1* under photoperiod-unfavorable conditions (**Figure 7B**) eventually make the flowering with various degrees in LD13 + 4B (10, 20, 30, or 40) and LD13 + NI-4B (10, 20, or 30) (**Figure 2A**). Therefore, the photoperiodic flowering in chrysanthemum should be co-regulated by both florigen and anti-florigen, and the balanced synthesis of both of them determines the flowering response to photoperiodic light treatments.

Flowering-related gene expression investigations in shoot apexes and leaves have improved our understanding of how to control chrysanthemum flowering. Currently, *CsFTL1*, *CsFTL2*, and *CsFTL3* were identified-three chrysanthemum orthologues of *FT* in *Chrysanthemum seticuspe*, and the expression of *CsFTL3* was observed as a key regulator in chrysanthemum photoperiodic flowering (Oda et al., 2012). In the flower-inductive SD conditions, *CsFTFL3* up-regulated the floral-identity genes to promote flowering events occurring in the SAM (Oda et al., 2012). Moreover, overexpressed *CsFTL3* induced SDP chrysanthemum flowering in LD environments, indicating that *CsFTL3* has the potential to induce chrysanthemum flowering under photoperiod-unfavorable conditions. In the present study, after 60 days of photoperiodic treatments, *CmFTL3* with two photoreceptor genes—*CmPHYA* and *CmCRY1* in *Chrysanthemum morifolium* were highly expressed in SD10 + 4B, SD10 + NI-4B, and LD13 + 4B treatments and gradually promoted from 10B to 30B and inhibited by 40B. Moreover, they were generally lower in LD13 + NI-4B and barely expressed in non-flowered LD13 and LD13 + NI-4B (40) treatments (**Figure 7B**). Moreover, during the temporal expression patterns of flowering-related genes in chrysanthemum leaves, the expression of *CmFTL3* was down-regulated by the light-signalling-mediated gene *CmPHYB* (**Figure 8**). All of our results indicate the chrysanthemum photoperiodic flowering is correlated with the photoreceptor-mediated control.

### Chrysanthemum flowering in response to co-regulation of photoperiod- and sucrose-mediated regulation under various intensities of supplemental or night-interruptional blue light in photoperiodic treatments

Sugar signaling is crucial for a variety of developmental activities, such as controlling the induction of flowers (Yu et al., 2013; Moghaddam and Ende, 2013; Yang et al., 2013). The most typical sugar produced by plants is sucrose, which is a more transportable substance due to its more stable molecule than either of its monosaccharide components, glucose or fructose. When a leaf of a photosensitive plant is exposed to a single inductive photoperiod, the amount of leaf sucrose increases quickly (Houssa et al., 1991). In *Arabidopsis*, feeding the aerial portion of dark-grown plants sucrose encourages flowering (Ohto et al., 2001). Sucrose produced by photosynthetic activity in *Arabidopsis* plants growing in LD causes *miR156* (Yu et al., 2013; Yang et al., 2013) to be down-regulated, which leads to the accumulation of the SQUAMOSA PROMOTER BINDING PROTEIN-LIKE gene (*SPL*) transcript and increases the expression of *FT* (Wahl et al., 2013). A photoperiod-based transcriptional regulation of florigen cannot fully explain the blooming response of chrysanthemum because the transition from vegetative to reproductive development is closely regulated (Higuchi et al., 2013). In SDP *Chrysanthemum morifolium*, gibberellin and photoperiod pathways cooperate to induce blooming in ‘Floral Yuuka’ plants growing in short days. Additionally, plants treated with sucrose growing under either an SD or an SD+NB regime continued to accumulate *CmFTL2* transcript (Sun et al., 2017), indicating that in ‘Floral Yuuka’, both photoperiod and sucrose affect *CmFTLs* transcription. Sugar signaling is more significant under LD circumstances (Ren et al., 2016). These findings imply that in ‘Floral Yuuka’ plants grown on short days, sucrose signaling may not play a significant role in floral induction. In our results, *CmFTL2* were highly expressed in the leaves under flower inductive conditions (LD13 + 4B (10, 20, 30, or 40) and LD13 + NI-4B (10, 20, or 30)) followed by all SD conditions, but really poor in non-flowered LD13 and LD13 + NI-4B (40) treatments. Moreover, LD13 + 4B (10, 20, or 30) also resulted the higher expression of florigen genes (like *CDM111*, *CmAFL1*, and *CmFL*) (**Figure 7B**). And, the carbohydrate was also significantly higher in these treatments (**Figures 5C, D**). Furthermore, the temporal expression pattern of *CmFTL2* was similar to *CmPHYA* and *CmCRY1*, but in contrast to *CmPHYB* (**Figure 8**). The florigen complex is most likely affected by *CmFTL2* rather than the anti-florigen complex. It is still unknown how *CmFTL2* functions in the regulatory network as well as the specifics of the cross-talk between photoperiod and endogenous sucrose levels during floral induction. The photoperiod- and sucrose-mediated regulation of blooming time in chrysanthemums may be significantly influenced by the sugar-induced *CmFTL2* pathway.

## Conclusions

In a conclusion, the S-B or NI-B interacts with the intensity and photoperiod differently affecting the morphology and physiology of the chrysanthemum plant. Generally, 30 μmol m^−2^ s^−1^ PPFD S-B (in both SD and LD conditions) and NI-B (in SD conditions) were more effective in promoting growth, flowering, and the expression of florigen genes. However, 40B leads to the unbalanced expression of florigen or anti-florigen genes, resulting in flowering inhibition, although it also can obviously improve morphological and physiological traits. Our current findings suggest the photoperiodic flowering of SDP chrysanthemum by the co-regulation of carbohydrate accumulation produced by photosynthetic carbon assimilation and the expression of florigen and anti-florigen genes mediated by differential photoreceptors in response to the intensity of S-B or NI-B. Moreover, *CmTFL1* affects chrysanthemum morphologies by promoting branching and leafing but inhibiting flowering. And the photoperiod- and sucrose-mediated regulation of flowering time in chrysanthemums may be significantly influenced by the sugar-induced *CmFTL2* pathway. Furthermore, the SDP chrysanthemum under LD13 + 4B (10, 20, 30, or 40) and LD13 + NI-4B (10, 20, or 30) conditions might be regulated by the LD florigen-like gene *CmFTL1* and sugar-induced *CmFTL2* eventually make the flowering. Further studies, the molecular mechanisms involved in these photoreceptor-mediated photoperiodic regulatory systems need to be explored in depth.

## Data availability statement

The original contributions presented in the study are included in the article/supplementary materials. Further inquiries can be directed to the corresponding author.

## Author contributions

JY and BRJ conceived and designed the study. JY and JS performed the experiments. JY analyzed the data and drafted the manuscript. JY and BJ edited and finalized the manuscript. All authors read and approved the submitted version.

## Funding

This research received no external funding. Jingli Yang and Jinnan Song were supported by the BK21 Four Program, Ministry of Education, Republic of Korea.

## Conflict of interest

The authors declare that the research was conducted in the absence of any commercial or financial relationships that could be construed as a potential conflict of interest.

## Publisher’s note

All claims expressed in this article are solely those of the authors and do not necessarily represent those of their affiliated organizations, or those of the publisher, the editors and the reviewers. Any product that may be evaluated in this article, or claim that may be made by its manufacturer, is not guaranteed or endorsed by the publisher.
